# Stomach-staggers in Horses

**Published:** 1898-09

**Authors:** H. W. Smith

**Affiliations:** Pass Christian, Miss.


### STOMACH-STAGGERS IN HORSES.

By H. W. Smith, V.S.,
PASS CHRISTIAN, MISS.

The spring and forepart of the summer in this section were exceedingly dry, and what little farming or gardening that was done was as near a total failure as could be. It was noticed by many that, although the grass and many other things were suffering, the trees, shrubs, and many under plants were of an unusual greenness. When the rains did set in they were very heavy, and two or three weeks afterward flatulent colic was rife and some deaths occurred. The cause of these cases was laid to the Mexican clover, which is abundant here, and used for haying; yet all grasses grew rapidly. Soon two bovine cases occurred which were spinal meningitis. Then reports came to me from distances that horses were dying of a strange disease, and from descriptions I concluded that the cases were those of the disease aforementioned. A case occurred right here, and I saw it. It was staggering around the yard in a half-dazed manner; the pulse below the normal, and the breathing appeared to be increased. When touched ever so lightly over the spine the horse would bend nearly to the ground; the upper lip was curiously elongated. This I thought was meningitis. The next day there was a yellow, oily, and very offensive discharge from the nostrils, and a tinkling sound could be heard at the base of the windpipe. It was, in my opinion, a case of impaction of the stomach, and before he died he tore everything to pieces.

In the next case the delirium was the first symptom. There have since been many deaths, but paralysis in none. Now, on Sunday, I saw a horse that went down quickly; the symptoms were much the same as in other cases; the pulse was 38; respirations 20; temperature appeared to be normal; could drink; pulsation in jugular vein very pronounced, and grinding of the teeth nearly continuous. The next day the discharge from the nose was very offensive; the tongue was as limp as a rag; the power to move the jaws was gone, and the mouth was full of maggots. This horse passed manure that contained beets, pieces of dry Bermuda stems, and fine straws in abundance, that were from an inch to two inches in length, oak-leaves that had not a mark of the teeth, and pieces of paper-like bark of pine of the size of a silver dollar. Could move the limbs, and offered resistance when the tail was raised, and was conscious of the movement of the hand before the eyes, yet was unable to stand.

All the cases much resemble meningitis, but are not, and are, to my mind, nothing but stomach staggers. There have been many cases along this coast, nearly every one of which has been fatal. Is there any successful way to treat these cases?

[These cases correspond in every particular to the many reported enzoötic outbreaks of the so-called cerebro-spinal meningitis. The condition of ingesta referred to indicates loss of deglutition of liquids many hours before the animals are unable to swallow solids. Will any of our readers kindly suggest further light and treatment recommended.—EDITOR.]

---

Present experiments by the Bureau of Animal Industry in dipping cattle for removal and destruction of the ticks (*Boophilus bovis*) are with a refined paraffin-oil, associated with other products. Recently eleven carloads of cattle were dipped at Fort Worth, Texas, under the supervision of Dr. Rice P. Steddom, of the Bureau of Animal Industry, and shipped by order of the Illinois State Board of Agriculture and Secretary Wilson, of the United States Department of Agriculture, to Rockford, Ill., and there placed on several farms with native cattle. So far the experiment is reported as a complete success, as the dip either removed or killed every tick on the dipped cattle. Should further experiments prove this conclusively, the cattle business in the Southwest will be revolutionized.

A better appreciation and closer adherence to regulations are reported this year along the Texas-fever quarantine-lines. Texas fever has been somewhat prevalent in Kansas, taken north by Southern cattle too late in the season.

